# MiR-218 affects hypertrophic differentiation of human mesenchymal stromal cells during chondrogenesis via targeting *RUNX2*, *MEF2C*, and *COL10A1*

**DOI:** 10.1186/s13287-020-02026-6

**Published:** 2020-12-10

**Authors:** Svitlana Melnik, Jessica Gabler, Simon I. Dreher, Nicole Hecht, Nina Hofmann, Tobias Großner, Wiltrud Richter

**Affiliations:** 1 Research Centre for Experimental Orthopaedics, Heidelberg University Hospital, Heidelberg, Germany; 2 Clinic for Orthopaedics and Trauma Surgery, Heidelberg University Hospital, Heidelberg, Germany

**Keywords:** miR-218, Mesenchymal stromal cells (MSC), Chondrocyte hypertrophy, Osteoarthritis (OA), *MEF2*C, *RUNX2*, Collagen type X

## Abstract

**Background:**

Human mesenchymal stromal cells (MSC) hold hopes for cartilage regenerative therapy due to their chondrogenic differentiation potential. However, undesirable occurrence of calcification after ectopic transplantation, known as hypertrophic degeneration, remains the major obstacle limiting application of MSC in cartilage tissue regeneration approaches. There is growing evidence that microRNAs (miRs) play essential roles in post-transcriptional regulation of hypertrophic differentiation during chondrogenesis. Aim of the study was to identify new miR candidates involved in repression of hypertrophy-related targets.

**Methods:**

The miR expression profile in human articular chondrocytes (AC) was compared to that in hypertrophic chondrocytes derived from human MSC by microarray analysis, and miR expression was validated by qPCR. Putative targets were searched by in silico analysis and validated by miR reporter assay in HEK293T, by functional assays (western blotting and ALP-activity) in transiently transfected SaOS-2 cells, and by a miR pulldown assay in human MSC. The expression profile of miR-218 was assessed by qPCR during in vitro chondrogenesis of MSC and re-differentiation of AC. MSC were transfected with miR-218 mimic, and differentiation outcome was assessed over 28 days. MiR-218 expression was quantified in healthy and osteoarthritic cartilage of patients.

**Results:**

Within the top 15 miRs differentially expressed between chondral AC versus endochondral MSC differentiation, miR-218 was selected as a candidate miR predicted to target hypertrophy-related genes. MiR-218 was downregulated during chondrogenesis of MSC and showed a negative correlation to hypertrophic markers, such as *COL10A1* and *MEF2C*. It was confirmed in SaOS-2 cells that miR-218 directly targets hypertrophy-related *COL10A1*, *MEF2C*, and *RUNX2*, as a gain of ectopic miR-218 mimic caused drop in MEF2C and RUNX2 protein accumulation, with attenuation of *COL10A1* expression and significant concomitant reduction of ALP activity. A miR pulldown assay confirmed that miR-218 directly targets *RUNX2*, *MEF2C* in human MSC. Additionally, the gain of miR-218 in human MSC attenuated hypertrophic markers (*MEF2C*, *RUNX2*, *COL10A1*, *ALPL*), although with no boost of chondrogenic markers (GAG deposition, *COL2A1*) due to activation of WNT/β-catenin signaling. Moreover, no correlation between miR-218 expression and a pathologic phenotype in the cartilage of osteoarthritis (OA) patients was found.

**Conclusions:**

Although miR-218 was shown to target pro-hypertrophic markers *MEF2C*, *COL10A1*, and *RUNX2* in human MSC during chondrogenic differentiation, overall, it could not significantly reduce the hypertrophic phenotype or boost chondrogenesis. This could be explained by a concomitant activation of WNT/β-catenin signaling counteracting the anti-hypertrophic effects of miR-218. Therefore, to achieve a full inhibition of the endochondral pathway, a whole class of anti-hypertrophic miRs, including miR-218, needs to be taken into consideration.

## Background

Human bone marrow-derived mesenchymal stromal cells (MSC) are a promising cell source for tissue engineering of cartilage defects due to their multi-lineage differentiation capacity, including chondrogenic potential [1, 2]. However, in vitro MSC-generated chondrocytes by a classical TGFβ-driven chondrogenesis protocol are characterized by unstable chondrocyte-like phenotype and undergo hypertrophic degeneration [3, 4]. Hypertrophy is a terminal stage of chondrocyte differentiation and also is one of the prominent characteristics of osteoarthritis (OA) [5]. Expression of cartilage-specific collagen type II and proteoglycans in MSC-derived chondrocytes is accompanied by induction of hypertrophic markers, such as collagen type X and alkaline phosphatase (ALP) activity [3, 6], both associated with the differentiation cascade leading to mineralization and endochondral bone formation after ectopic transplantation. On the contrary, mature chondrocytes derived from the cartilage (defined as articular or hyaline chondrocytes, AC), cultured under similar conditions, remain negative for collagen type X and ALP and form stable non-calcified ectopic cartilage transplants [3].

The molecular mechanisms that are involved in the induction and regulation of chondrocyte hypertrophy are complex and remain poorly understood. Hypertrophic differentiation of chondrocytes is promoted or inhibited by multiple factors involved in different cell signaling pathways, including insulin-like growth factors (IGFs) [7], parathyroidhormone-related peptide (PTHrP) [8], Indian hedgehog (IHH) [9], fibroblast growth factors (FGFs) [10], canonical and non-canonical WNT signaling [11], and transforming growth factor β (TGFβ) family members with bone morphogenetic proteins (BMPs) [12]. At a transcriptional level, Runt-related transcription factors 2 (*RUNX2*) and myocyte enhancer factor-2C (*MEF2C*) have been implicated as key transcription factors regulating chondrocyte hypertrophy, as they drive expression of the terminal differentiation markers, such as collagen type X [13–15], metalloproteinases MMP3 [16] and MMP13 [17–19], integrin-binding sialoprotein (IBSP) [20, 21], Indian hedgehog (IHH) [20, 22], and ALP [3, 20, 23, 24]. Studies in mouse models have demonstrated that several evolutionarily conserved Runx2 and Mef2c binding sites were found at the murine *Col10a1* enhancer and promoter [15, 25], suggesting a direct regulatory role these transcription factors play in murine *Col10a1* gene expression. *Mef2c* is expressed in pre-hypertrophic and hypertrophic chondrocytes, and its genetic deletion or expression of a dominant-negative *Mef2c* mutant in endochondral cartilage impairs hypertrophy [13]. Additionally, *Mef2c* mutant mice showed a dramatically diminished expression of *Runx2*, suggesting that, epistatically, Runx2 acts downstream of Mef2c [13]. Remarkably, although it was believed that Runx2 and Runx3 play redundant roles in mouse chondrocyte maturation [9, 26], in human MSC, RUNX3, but not RUNX2, has been demonstrated to be the key transcription factor, together with MEF2C, driving hypertrophy and regulating the endochondral pathway in MSC, whereas RUNX2 has been rather assigned as an anti-chondrogenic factor [20].

Numerous studies (reviewed in [27]) suggest that microRNAs (miRs) play an important role in chondrocyte biology by suppressing their target gene expression at the post-transcriptional level. MicroRNAs are 22 nucleotide-long noncoding RNAs that directly bind to target mRNAs in a sequence-complimentary manner to facilitate the degradation of their target transcripts. To date, there is limited knowledge of miRs that are directly involved in post-transcriptional regulation of hypertrophy-related mRNAs during chondrogenesis. It has been suggested that a number of different miRs might target *Runx2* in the murine chondrogenic cell line ATDC5 [28]. Of those, miR-218 (other name: hsa-miR-218) was demonstrated to directly target *RUNX2* in human osteogenic dental stem cells (DSC) [29] and *HPGD* (15-hydroxyprostaglandin dehydrogenase) in rabbit synovium-derived MSC promoting their chondrogenic differentiation [30]. Additionally, miR-29b in murine MSC has been demonstrated to promote the hypertrophic phenotype [31], and miR-381 resembled anti-hypertrophic properties in the human chondrosarcoma cell line SW1353 [32].

In our previous study, we have identified a set of differentially expressed miRs during in vitro chondrogenesis of human MSC and defined miR clusters specific for different stages of chondrogenic differentiation (prechondrocytes, chondroblasts, chondrocytes, and hypertrophic chondrocytes) [33]. However, it was not clear whether among the identified miR clusters were those related to hypertrophy onset. Also, no mRNA targets were characterized by those differentially expressed miRs.

In the present study, we addressed the question which miRs are directly involved in post-transcriptional regulation of hypertrophy-related genes. For this, human bone marrow-derived MSC were subjected to TGFβ-driven chondrogenesis resulting in hypertrophic chondrocytes. Their miR expression profile was determined by microarray analysis and compared to that of re-differentiated non-hypertrophic human articular chondrocytes (AC). We identified miR-218 as the best candidate to target hypertrophic genes and validated its function during human MSC chondrogenesis. This allowed us to draw conclusions about a role of this miR in the hypertrophic phenotype regulation during chondrogenic differentiation of human MSC.

## Material and methods

### Cell lines, isolation of MSC and AC, and cell expansion

Human cell lines: embryonic kidney epithelial HEK293T and bone osteosarcoma SaOS-2 were from the American Type Culture Collection (ATCC), and cells were grown in DMEM (Gibco) supplied with 10% fetal calf serum (FCS, Sigma), 100 I.U./mL penicillin, 100 μg/mL streptomycin (Biochrome).

MSC were isolated from fresh bone marrow aspirates of patients undergoing elective bone surgery (*N* = 17 donors). AC were isolated from knee cartilage of *tibia plateau* that was removed due to OA (*N* = 13 donors), or from healthy donors (*N* = 21), either undergoing arthroscopic surgery of a knee due to trauma, or from deceased due to unrelated causes patients. The healthy articular cartilage was carefully removed from regions with no macroscopically evident degeneration and washed with phosphate-buffered saline (PBS), to avoid contamination by other cells.

The study was approved by the local ethics committee (Medical Faculty of the University of Heidelberg), and the informed consent was obtained from all the patients participated in the study, according to the 1964 Declaration of Helsinki, updated in 2000. All cells used for experiments in the study were from HIV-, HBV-, and HCV-negative donors.

MSC population was isolated, as described before [12]. In brief, the mononuclear cell fraction was separated from bone marrow aspirates by Ficoll-Paque™ density-gradient and seeded at density of 1.25 × 10^5^ cells/cm^2^ into 0.1% gelatin-coated culture flasks in expansion medium (DMEM high glucose, 12.5% FCS, 100 I.U./mL penicillin, 100 μg/mL streptomycin, 2 mM l-glutamine, 1% non-essential amino acids, 0.1% β-mercaptoethanol (all from Life Technologies), and 4 ng/mL FGF-2 (Active Bioscience, Germany)). After 24 h, non-adherent cells were removed by washing with PBS. The medium was replaced 3 times a week, and cells were expanded until passage 3. For passaging, once cells reached 80% of confluence, they were detached with trypsin/EDTA and plated at density 5000 cells/cm^2^.

For articular chondrocyte isolation, cartilage pieces were minced, and chondrocytes were released by overnight digestion with collagenase B (Roche, Germany) and hyaluronidase (Sigma-Aldrich, Germany), as described before [34]. Chondrocytes were expanded in DMEM medium containing 10% FCS, 100 IU/mL penicillin, 100 μg/mL streptomycin, at 37 °C, 6% CO_2_. The medium was changed twice per week, and cells were expanded until passage 2.

### Chondrogenic differentiation (MSC) and re-differentiation (AC)

For chondrogenic differentiation (MSC) or re-differentiation (AC), at the 3rd (MSC) or 2nd (AC) passages, 5 × 10^5^ of MSC or AC were resuspended in chondrogenic medium (DMEM high glucose, 0.1 μM dexamethasone, 0.17 mM ascorbic-acid 2-phosphate, 5 mg/ml transferrin, 5 ng/ml sodium selenite, 1 mM sodium pyruvate, 0.35 mM proline, 1.25 mg/ml bovine serum albumin (BSA), 100 I.U./mL penicillin, 100 μg/mL streptomycin, 5 mg/ml insulin (Sanofi-Aventis, Germany), and 10 ng/ml TGF-β1 (PeproTech, Germany)). Cells were centrifuged at 500 g for 5 min, or allowed to self-aggregate without centrifugation, to generate high-density pellets. Pellets were cultured for 3, 4, or 6 weeks at 37 °C, 6% CO^2^ with medium change 3 times a week.

### MicroRNA microarray analysis

Five pellets per donor (MCS, day 42, *N* = 3; AC, day 21, *N* = 1) were pooled, mechanically minced in a polytron, and total RNA was isolated by guanidinium thiocyanate/phenol extraction with peqGOLD Trifast (PeqLab, Germany). Isolated total RNA was used for miR expression analysis with an Agilent Human microRNA Microarray v16.0 (1349 miRs; Agilent Technologies, USA). The quality control of total RNA, labeling, array hybridization, and microarray scanning was performed at the DKFZ Genomics Core Facility (Heidelberg, Germany). Microarray scanning was done using an iScan array scanner. Data extraction was done for all beads individually, and outliers were removed when the absolute difference to the median was greater than 2.5 times MAD (mean of absolute deviation) (2.5 Hampel’s method). All remaining bead level data points were then quantile normalized. As a test for significance, the Student’s *t* test was used on the bead expression values of the two groups of interest, and Benjamini-Hochberg correction [35] was applied to the complete set of *p* values of all ProbeIDs on the chip. The average expression value was calculated as the mean of the measured expressions of beads, together with the standard deviation of the beads.

### In silico target prediction analysis and KEGG pathway analysis

MicroRNA target prediction tools TargetScan, MiRWalk, and miRanda were applied to identify putative mRNA targets for miR-218. Putative mRNA targets with strong mirSVR (microRNA score vector regression) downregulation score (− 0.4, http://www.microRNA.org) suggested by all used prediction tools were included in further consideration and analysis. For systematic analysis of GO (gene ontology) terms and KEGG (Kyoto Encyclopedia of Genes and Genomes) signaling pathways of putative mRNA targets suggested by the miR prediction tools, the DAVID (Database for Annotation, Visualization, and Integrated Discovery) gene functional classification tool was applied, with a stringency set at the highest level [36].

### RNA extraction and quantitative reverse-transcriptase PCR (qRT-PCR)

Total RNA was isolated from cells and pellets using a standard guanidiniumthiocyanate/phenol extraction protocol with peqGOLD TriFast™ reagent (Peqlab, Erlangen, Germany). Total RNA from cartilage tissue was isolated, as described before [37, 38]. Polyadenylated mRNA was isolated using oligo d(T)-coupled magnetic beads (Dynabeads, Dynal, Invitrogen GmbH, Karlsruhe, Germany), according to the manufacturer’s instruction. For the first-strand cDNA synthesis, 20 ng of mRNA was used with reverse transcriptase (Omniscript®, Qiagen, Hilden, Germany) and oligo-d(T) primers. Quantitative reversed transcriptase PCR (qRT-PCR) was performed using the SYBR green I mix (ThermoScientific, Rockford, USA) and gene-specific primers (Table S1) with StratageneMx3000P (Agilent Technologies, Böblingen, Germany).

For the quantification of microRNAs expression, 10 ng of total RNA was used for cDNA synthesis with the TaqMan®MicroRNA Reverse Transcription Kit (ThermoFisher Scientific). Relative expression levels were determined by qRT-PCR using the miR detection method from the Applied Biosystems (MicroRNA Reverse Transcription Kit) combined with the TaqMan MicroRNA Assays, using primers for U6 (Assay ID:0001973) and hsa-miR-218-5p (Assay ID: 000521). Expression levels of mRNA and miR were calculated using 2-ΔΔCT method [39] and normalized to housekeeping genes Actin Beta (*ACTB*), Hypoxanthine-guanine phosphoribosyltransferase (*HPRT*), Ribosomal Protein L13 (*RPL13*), and Cleavage and Polyadenylation Specific Factor 6 (*CPSF6)* (for mRNAs), or U6 (Small Nuclear Ribonucleoprotein U6, *SNRNU6)* (for miRs).

### 3′UTR luciferase reporter assay

The 3′UTR of *COL10A1* and *MEF2C* containing a seed region for miR-218 were amplified from genomic DNA by PCR. The 3′UTR fragment was cloned into SpeI and HindIII restriction sites of pMIR-REPORT Luciferase vector (ThermoFisher Scientific). The 3′UTR of *RUNX2* containing a seed region for miR-218, as well as all mutated sequences, were chemically synthesized (GeneArt, Germany). Primers used for this assay are listed in Table S2.

For miR gain-of-function experiments, microRNA mimics and the miR-218 inhibitor (anti-miR-218) were purchased from Ambion (mirVana: MC10328, MH10328, 4464058).

HEK293T cells, seeded a day before transfection at a density 5 × 10^4^ cells/well, were cultured on 24 well plates. Co-transfection of 50 nM of a selected miR mimic combined with 250 ng of a corresponding tested reporter construct, together with 250 ng of a normalization control β-Gal vector (ThermoFisher Scientific), was carried out using Lipofectamine 2000 reagent (Invitrogen, Germany). Luciferase activity was measured 48 h after transfection with Victor3 Multilabel Counter 1420–042 using the Luciferase Reporter Assay Kit (Promega, USA). Luciferase intensity signals were normalized to the β-Galactosidase activity in the same sample. Three independent experiments with six biological replicates were performed for this assay.

### Ectopic overexpression of miR-218 in SaOS-2 and MSC cells

For miR overexpression in SaOS-2 and MSC cells, electroporation (in case of SaOS-2) (Neon, ThermoFisher Scientific), or transfection with Dharmafect1 (Dharmacon) (in case of SaOS-2 or MSC cells) were carried out, according to the manufacturers’ instruction. In brief, for electroporation, 1 × 10^6^ cells mixed with 10 μM of miR mimic in 100 μl R-buffer were electroporated with two pulses at 1200 V for 15 ms. For a reverse method of transfection using Dharmafect1, 10^6^ cells (SaOS-2 or MSC) resuspended in 100 μl of DMEM medium containing no additives, were transfected with 50 nM of miR mimic, according to the manufacturer’s protocol. In case of MSC, transfected cells were transferred in chondrogenic medium and subjected to chondrogenic differentiation for 28 days, as described above. SaOS-2 cells were harvested 72 h after transfection for Western blotting and qRT-PCR analyses.

### MiR pulldown assay

For the miR pulldown assay, MSC at passage 3 were harvested, washed 2 timed in ice-cold PBS and lysed (100 μl of buffer per10^6^ cells) in RNase-free lysis buffer containing 10 mM HEPES (pH 7.9), 10 mM KCl, 1.5 mM MgCl_2_, 10 mM DTT, 10 mM PMSF, 1/100 of Halt™ protease and phosphatase inhibitor-cocktails (PIC) (ThermoFisher Scientific), 1/100 of RNaseOUT (Invitrogen) for 30 min on ice. The cell lysate was cleared by centrifugation at 5000 g for 5 min, and the concentrations of KCl and MgCl_2_ were adjusted to 100 mM and 5 mM, correspondingly. NP40 was added to 0.5%, and the cell lysate was aliquoted, with preservation of an aliquot for the input assessment. To each aliquot, 1 μM of either miR-218 or a control non-targeting (NC) biotin-labeled miR mimic was added (biotin hsa-miR-218-5p miRCURY LNA Premium miRNA Mimic: YM00471984-BDI, and Negative Control miRCURY LNA miRNA Mimic: YM00479902-BDI, Qiagen) and incubated for 4 h at 4 °C. Next, 20 μl of pre-blocked (for 1 h in lysis buffer containing 250 μg RNase-free BSA (NEB) and 100 μg yeast tRNA (ThermoFisher Scientific)) streptavidin magnetic beads (Roche) were added to the cell lysate aliquots and incubated overnight. Then, beads were washed 5 times in RNase-free IP buffer (50 mM Tris-HCl (pH 7.4), 100 mM KCl, 5 mM MgCl_2,_ 0.5% NP40, 10 mM DTT, 10 mM PMSF). Total RNA was extracted from beads using peqGOLD TriFast™ reagent (Peqlab, Erlangen, Germany) and processed for RNA analysis by quantitative reverse transcriptase polymerase chain reaction (qRT-PCR), as described above, omitting the isolation of polyadenylated mRNA using oligo d(T)-coupled magnetic beads step.

### Western blotting (WB)

Cells and pellets were lysed in lysis buffer containing 50 mM Tris-HCl (pH 7.4), 150 mM NaCl, 1% Triton X-100, for 5 min on ice, with a preliminary grinding step (in case of pellets) using Powerful ball mill Retsch MM400 (2 rounds for 2 min, 30 Hz). For analyses of cytosolic β-catenin, cell extracts were prepared in Saponin lysis buffer (0.05% saponin, 1 mM MgCl_2_, 50 mM Tris-HCl (pH 7.4), 150 mM NaCl, 2.75 mM KCl, 0.1 mM β-mercaptoethanol) [40]. Cell lysates were cleared by centrifugation, and proteins were resolved by SDS-PAGE, blotted onto a nitrocellulose membrane and analyzed by WB. Following antibodies were used: MEF2C (Cell Signaling, D80C1), RUNX2 (MBL, D130-3), β-catenin (BD Transduction Laboratories, 610153), α-tubulin (ThermoFisher Scientific, MS-581-P0), and β-actin (AC-15, GeneTex, USA).

### ALP activity assay

ALP activity was measured by colorimetric assay using multi-mode plate reader FLUOstar Omega (BMG Labtech). For this, cell culture supernatants (5 per group or treatment), conditioned for 2 days, were collected and mixed with an equal volume (100 μl) of ALP substrate solution (10 mg/ml p-nitrophenylphosphate in 0.1 M glycine, 1 mM MgCl_2_, and 1 mM ZnCl_2_, pH 9.6). After incubation for 180 min, absorbance recorded at 405 nm was corrected for a signal at 490 nm, and enzyme activity was quantified using a standard curve built using serial dilutions of p-nitrophenol (Sigma Aldrich). Six independent experiments with two technical replicates were performed for this assay.

### Histology and immunohistochemistry

Pellets were fixed for 24 h in 4% formaldehyde, dehydrated in a graded isopropanol series, and paraffin-embedded. Thin sections (5 μm) were stained with Safranin O (0.2% in 1% acetic acid) using Certistain® fast green (0.04% in 0.2% acetic acid) for counter-staining, according to a standard procedure. Immunohistochemical detection of collagens, type II and type X, was performed as described [41]. Briefly, sections treated with 4 mg/mL hyaluronidase in PBS, pH 5.5, and 1 mg/mL pronase, then blocked with 5% BSA and stained with collagen type II antibody (clone II-4C11, ICN Biomedicals), or collagen type X antibody (clone X53, Quartett, Germany), followed by incubation with biotinylated goat anti-mouse antibody (1:500, Dianova), and subsequent detection with streptavidin alkaline-phosphatase fast red (ThermoFisher Scientific).

### Quantification of proteoglycan content (DMMB assay)

Proteoglycan content in the cartilaginous tissue was measured by DMMB (dimethyl-methylene blue) assay, as described before [42]. For this, pellets (*N* = 2 per donor) were harvested at day 28 of the chondrogenic induction, washed with PBS, and digested overnight in 1 ml of lysis buffer containing 50 mM Tris-HCl (pH 8.0), and 1 mM CaCl_2_ with 500 μg/ml Proteinase K (Roche, Mannheim, Germany) at 60 °C. Pellets digests (30 μl) were mixed with 200 μl of DMMB solution (38 μM dimethyl-methylene blue, Sigma, 40 mM glycine, 40 mM NaCl). Proteoglycan content was measured by spectrophotometry at 540 nm and quantified using a standard curve built with chondroitin sulphate substrate standard. The values were normalized to DNA amount in lysed cells measured using Quanti-iT PicoGreen dsDNA kit (Invitrogen, Eugene, USA). For this, 20 μl of the digested pellet sample were mixed with 80 μl TE buffer (200 mM Tris-HCl (pH 7.4), 20 mM EDTA) and PicoGreen solution, and fluorescence in samples was measured at 485/535 nm.

### Statistical analysis

Statistical analysis was performed using GraphPad (Prism) software, with application of either Mann-Whitney *U* test, or two-tailed Student’s *t* test; expression kinetic curves were tested with paired two-tailed Student’s *t* test; *p* values ≤ 0.05 were referred to as being significant.

## Results

### Identification and selection of differentially expressed miRs between hypertrophic and articular chondrocytes

**Fig. 1:**
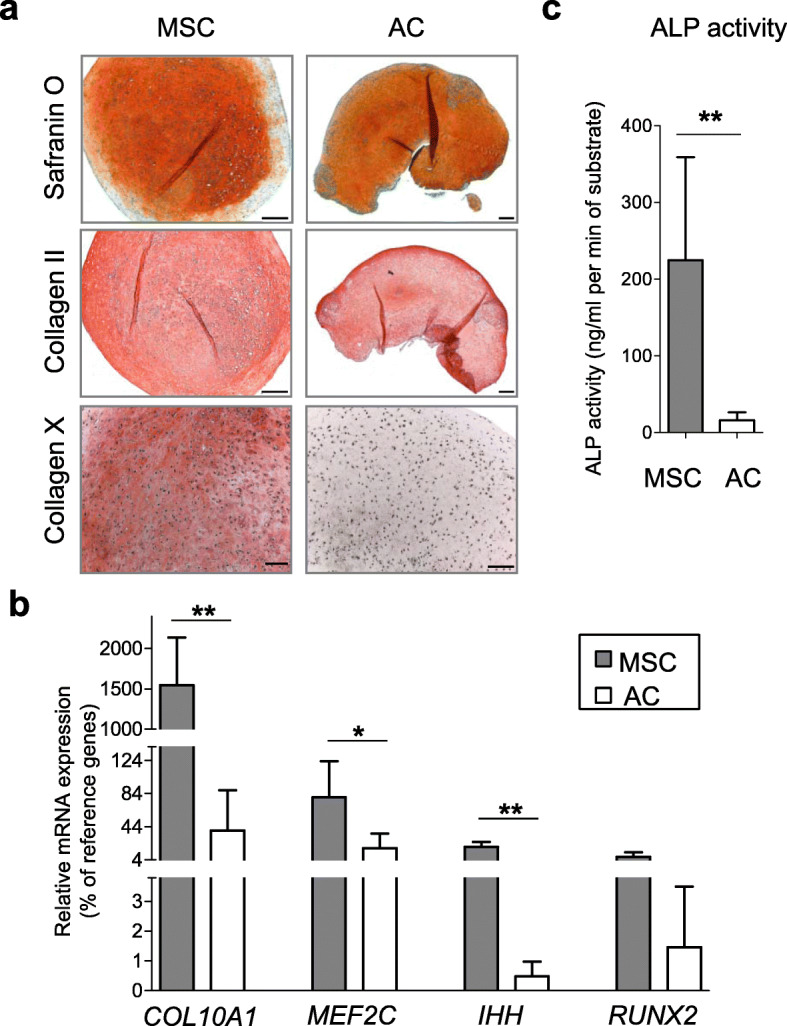
MSC, but not AC, undergo hypertrophy under chondrogenic conditions. MSC (*N* = 6 donors) or AC (*N* = 4 donors) were subjected to chondrogenic conditions and evaluated at the end of their chondrogenesis. **a** Representative images for Safranin O staining, and collagen type II or type X immunohistochemistry. Scale bar, 100 μm. **b** mRNA expression for indicated genes was measured by qRT-PCR, and values were normalized to three housekeeping genes (*ACTB*, *HPRT*, *RLP13*). **c** Alkaline phosphatase activity (ALP) measured in culture media from cell pellets. Mean values ±  SD are shown (MSC—gray, AC—white); *p ≤ 0.05, **p ≤ 0.01 (Mann-Whitney *U*)

To identify miRNAs that affect hypertrophy-associated signaling pathways, we used a direct comparison of miR expression profiles between hypertrophic and articular chondrocytes by microarray analysis. Upon chondrogenic in vitro differentiation in 3D culture, human bone marrow MSC undergo hypertrophy, whereas AC always remain negative for hypertrophic markers after re-differentiation at the same conditions. Indeed, when the human bone marrow MSC that underwent chondrogenic differentiation were compared to re-differentiated AC isolated from the knee cartilage, these two cell populations produced cartilaginous tissue cultures in pellets of distinct phenotypes (Fig. 1). Both resulting tissues showed pronounced staining with Safranin O, indicating a successful progression of chondrogenesis and proteoglycan deposition in both groups. This result was additionally supported by comparable levels of collagen type II immunostaining (Fig. 1a). However, in contrary to this, immunostaining for the hypertrophic marker collagen type X was positive in MSC and negative in AC, indicating that MSC, but not AC, underwent hypertrophic differentiation. This conclusion was also supported by mRNA expression data for *COL10A1* (Fig. 1b). Expression levels of other hypertrophy-associated markers, such as *MEF2C*, *IHH*, and *RUNX2*, were also significantly increased in MSC-derived chondrocytes in comparison to AC (Fig. 1b), and this was accompanied by significantly higher ALP activity in culture supernatants of pellet cultures formed by MSC in relation to a low enzyme activity for AC pellets (Fig. 1c). Collectively, these data highlight that chondrocytes derived from MSC or AC have different phenotypes in regard of hypertrophy, and these cells could serve as different chondrocyte cell populations to address whether specifically regulated microRNAs are related to induction of the hypertrophic phenotype.

Next, RNA samples isolated from pellets of chondrogenically differentiated MSC or AC were analyzed by a microRNA microarray. Among 1349 miR probes tested, 115 miRs showed more than 2-fold differences in their expression levels between MSC versus AC, with the top 15 reaching over 60-fold changes between MSC and AC (Table 1). These top 15 differentially expressed miRs were then subjected to in silico analysis with application of a single criterion: their putative mRNA targets had to be involved in regulation of hypertrophic pathways. Three microRNA target prediction tools: TargetScan, MiRWalk, and miRanda were utilized to identify putative mRNA targets. Among these 15 candidates, miR-218 was predicted to potentially target the bona fide hypertrophy markers *MEF2C*, *RUNX2*, and *COL10A1* with a substantial miRSVR (microRNA score of vector regression). MirSVR computed by http://www.microRNA.org tool uses vector regression (SVR) to evaluate a wide range of features allowing prediction and ranking of multiple putative microRNA target sites’ efficiencies. It serves as a measure of the likelihood that a given miR interacts with a certain sequence of mRNA target. It takes into account a secondary structure accessibility of the site, its conservation that affect miR-mRNA pairing, and a score lower than − 0.4 is generally regarded as a significant one (Table 2).

**Table 1:** Top 15 differentially expressed microRNAs between re-differentiated articular chondrocytes (AC) and hypertrophic chondrocytes produced from MSC, sorted according to their regulation factor

miR	AC	MSC	Factor
hsa-miR-181b	0.1	280	2802
hsa-miR-181a	0.1	144	1443
hsa-miR-513a-5p	0.1	37	375
hsa-miR-224	0.1	33	334
hsa-miR-1208	0.1	23	227
hsa-miR-4327	0.1	22	218
hsa-miR-3188	0.1	19	187
**hsa-miR-218**	**0.1**	**19**	**185**
hsa-miR-145	0.1	16	156
hsa-miR-181a	20	2898	146
hsa-miR-34b	0.1	13	126
hsa-miR-449c	0.1	12	121
hsa-miR-1226	0.1	7	73
hsa-miR-449b	0.1	6	62
hsa-miR-4254	0.1	6	60

**Table 2:**
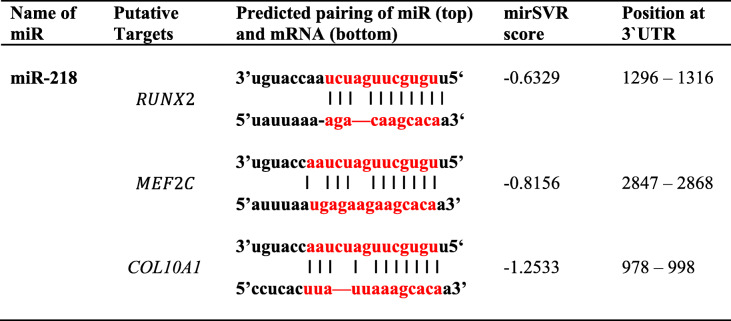
In silico analysis for annealing efficiencies between putative mRNA targets and miR-218

### Reversed correlation between miR-218 and hypertrophic marker expression kinetic profiles during MSC chondrogenesis

**Fig. 2:**
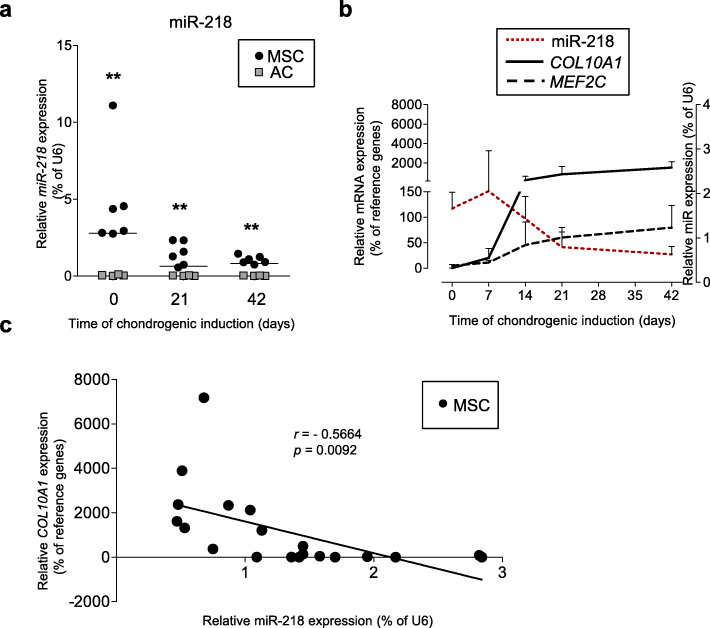
Negative correlation for gene expression kinetics between miR-218 and *MEF2C* or *COL10A1* in MSC during chondrogenic differentiation. **a** Differential expression of miR-218 between MSC (*N* = 6 donors, black circles) and AC (*N* = 4 donors, gray squares) was monitored at indicated time points during chondrogenic differentiation by qRT-PCR; vertical lines correspond to median values; **p ≤ 0.01 (Mann-Whitney *U*). **b** MSC (*N* = 4 donors) were subjected to chondrogenic induction, and expression of *MEF2C* (black-dotted line) and *COL10A1* (black line) mRNA and miR-218 (red-dotted line) was measured by qRT-PCR at indicated time points. Mean values ± SD are shown. **c** Correlation between *COL10A1* and miR-218 expression levels in MSC (*N* = 4 donors) was measured by qRT-PCR. Pearson correlation coefficient with its *p* value is indicated

Next, we validated the microarray results using qRT-PCR and also monitored the expression of miR-218 in MSC-derived chondrocytes at different stages of chondrogenesis and of re-differentiation of AC from four to six different donors. MiR-218 was already more expressed in expanded MSC in comparison to AC. Along the time-course of chondrogenesis, expression of miR-218 gradually declined: high expression levels at a start of chondrogenesis (day 0) later were reduced at days 21 and 42 (Fig. 2a), but always remained significantly above AC levels. Remarkably, the expression kinetic profiles for miR-218 and hypertrophic markers *MEF2C* and *COL10A1* showed a reverse regulation over 42 days of chondrogenesis (Fig. 2b). Additionally, expression of *COL10A1* and miR-218 showed a tight negative correlation (Pearson’s correlation coefficient *r = −* 0.5664 with *p* = 0.0092) during MSC chondrogenesis (Fig. 2c). These results imply a putative regulatory role of miR-218 in post-transcriptional regulation of *MEF2C* and *COL10A1* genes and its anti-hypertrophic function.

### MiR-218 directly targets hypertrophic marker genes

To understand the regulatory role of miR-218, we searched for additional mRNA targets, beyond of *MEF2C*, *RUNX2*, and *COL10A1*, using different web-based bioinformatics tools: TargetScan, MiRWalk, and miRanda. This resulted in 4434 high-confidence hits for miR-218, matched by all three algorithms. Gene Ontology (GO) and KEGG (Kyoto Encyclopedia of Genes and Genomes) pathway analyses revealed that among the functional and signaling pathways regulated by these putative targets, there was a significant enrichment for genes related to “signal transduction” (including known hypertrophy-regulating signaling pathways, e.g., WNT, TGFβ, HH (Hedgehog), and Thyroid hormone), “Signaling molecules and interaction” (including extracellular matrix (ECM) organization) and “Glycan biosynthesis and metabolism” (including glycosaminoglycan biosynthesis - chondroitin sulfate/dermatan sulfate) categories (Table 3). Altogether, this suggested a high complexity of putative miR-218 regulation mechanisms.

**Table 3:** Significantly enriched KEGG pathways associated with the predicted target genes for miR-218 (selected categories for “signal transduction,” “signaling molecules and interaction,” and “glycan biosynthesis and metabolism” identifiers)

KEGG pathway	Number of genes	*P* value
PI3K-Akt signaling pathway	106	3.7E−3
MAPK signaling pathway	86	3.5E−4
cAMP signaling pathway	72	1.2E−4
Calcium signaling pathway	67	7.9E−5
Hippo signaling pathway	56	4.3E−4
AMPK signaling pathway	49	1.6E−4
Signaling pathways regulating pluripotency of stem cells	45	2.9E−2
Wnt signaling pathway	43	5.3E−2
Thyroid hormone signaling pathway	40	1.1E−2
HIF-1 signaling pathway	38	1.1E−3
TNF signaling pathway	35	4.3E−2
NF-kappa B signaling pathway	30	3.3E−2
ECM-receptor interaction	28	8.6E−2
mTOR signaling pathway	27	4.3E−4
TGF-beta signaling pathway	27	9.3E−2
Hedgehog signaling pathway	11	9.3E−2
Glycosaminoglycan biosynthesis - chondroitin sulfate/dermatan sulfate	9	8.6E−2

**Fig. 3:**
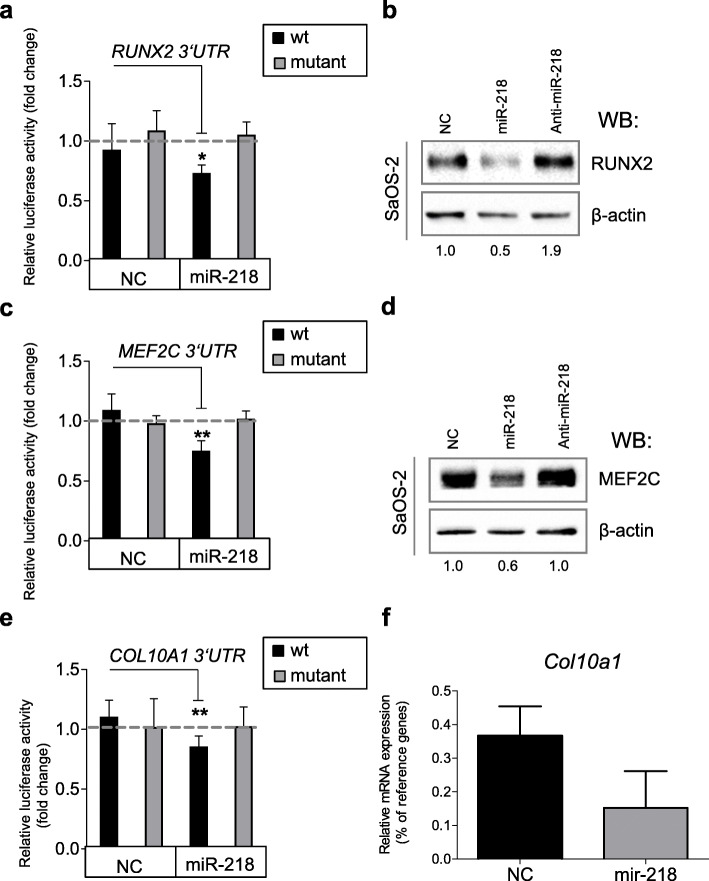
Hypertrophic marker genes are targets of miR-218. **a**, **c**, **e**): Luciferase assay in HEK293T cells conducted with pMIR-REPORT vector either containing wild-type (wt, black) or mutated (gray) 3′-UTR fragment of *RUNX2* (**a**), or *MEF2C* (**c**), or *COL10A1* (**e**) with a putative binding-side for miR-218. Non-targeting control miR mimic (NC) was used as a control. Gray-dotted lines indicate levels of binding for an empty pMIR-REPORT vector; *n* = 3. **b**, **d**, **f** SaOS-2 cells were transiently transfected with either miR-218, or with anti-miR-218 with non-targeting control (NC) mimics, and abundancies of RUNX2 (**b**) or MEF2C (**d**) proteins were detected by WB (numbers below indicate fold changes in comparison to NC control, *n* = 3 (RUNX2) or 5 (MEF2C)), or *COL10A1* mRNA expression was measured by qRT-PCR, *n* = 2 (**f**). Mean values ± SD are shown. **p ≤ 0.01 (Mann-Whitney *U*)

Since miR-218 directly targeted the transcription factor *RUNX2* in a number of mouse cells lines: osteoblasts (MC3T3-E1), chondrocytes (ATDC5), fibroblasts (NIH 3T3) [28], and human dental stem cells [29], we used it as a positive control mRNA target. A fragment of the *RUNX2* 3′UTR containing the predicted miRNA-218 seed region was cloned downstream of a CMV-driven firefly luciferase vector, and luciferase activity was measured after co-transfection with miR-218 mimic in HEK293T cells. To confirm the exact miR-218 binding region for *RUNX2*, a reporter vector with a mutated seed-region in the 3′UTR was used as a negative binding control. A non-targeting (NC) miR mimic was used as another negative control. Indeed, ectopic miR-218 mimic could significantly reduce activity of the tested *RUNX2* 3′UTR reporter (Fig. 3a) demonstrating that miR-218 directly regulates expression of *RUNX2*, as expected. This effect was abrogated by mutations introduced into the seed region for miR-218 in the 3′UTR of *RUNX2*. Also, no inhibiting effects were caused by the NC miR mimic (Fig. 3a). These results were additionally supported by Western blot analysis in SaOS-2 cells transiently transfected with miR-218 mimic, which revealed a substantial decline in RUNX2 protein accumulation due to forced elevation of miR-218 (Fig. 3b and Fig. S1), and a weak reverse effect was observed in case of anti-miR-218 mimic transfection.

Next, we engineered miR reporter plasmids for the putative recognition sites of *MEF2C* and *COL10A1* 3′UTRs. The luciferase activity for the wild-type *MEF2C* reporter was significantly reduced by the miR-218 mimic, while non-targeting (NC) miR mimic failed to reduce the reporter activity. A direct targeting effect of miR-218 mimic for *MEF2C* was additionally confirmed using mutated 3′UTR seed sequence for the *MEF2C* reporter (Fig. 3c). Again, this was verified in SaOS-2 cells transiently transfected either with miR-218 mimic, or with anti-miR-218, when decreased accumulation of MEF2C protein was observed due to a gain of miR-218 levels, and this effect went to the opposite direction upon transfection with anti-miR-218 (Fig. 3d and Fig. S1).

A similar inhibiting effect on luciferase activity was seen for a reporter containing a *COL10A1* 3′UTR with a putative seed sequence for miR-218 (Fig. 3e). Furthermore, in SaOS-2 cells, gain of miR-218 reduced *COL10A1* expression, although only by a trend due to very low endogenous levels of *COL10A1* mRNA in SaOS-2 cells (Fig. 3f and Fig. S1).

**Fig. 4:**
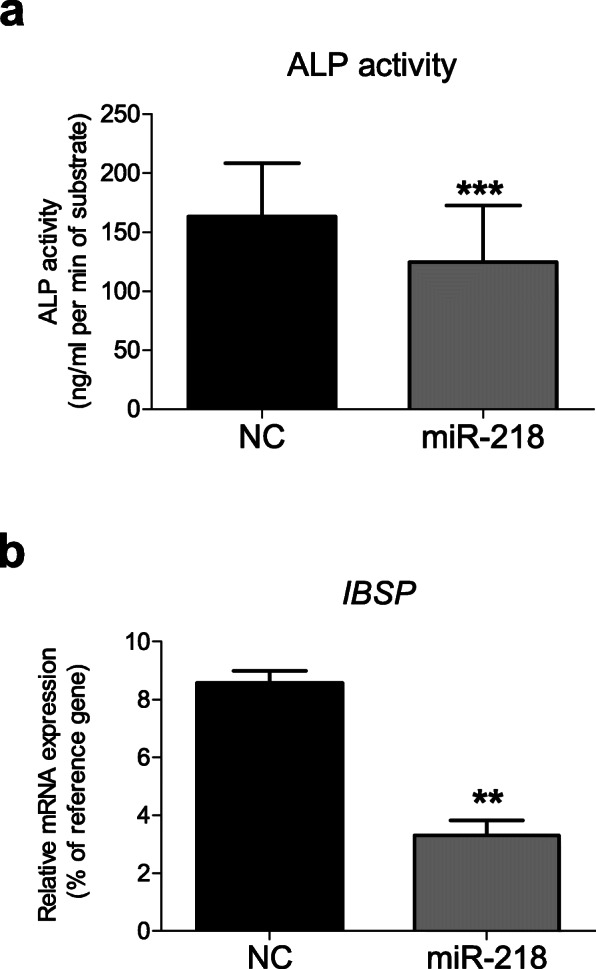
miR-218 reduces hypertrophic markers in SaOS-2 cells. SaOS-2 cells were transiently transfected with miR-218 mimic (gray) or with a non-targeting control miR mimic (NC, black), and ALP activity was measured in cell culture supernatants 48 h later (*n* = 8) (**a** mean values ± SEM are shown), or expression of *IBSP* mRNA was monitored by qRT-PCR (*n* = 3) (**b** mean values ± SD are shown); **p ≤ 0.01 and ***p ≤ 0.001; (two-tailed paired *t* test)

Collectively, these data indicated that miR-218 targets and downregulates a number of molecules involved in the endochondral pathway, including *RUNX2*, *MEF2C*, and *COL10A1.* Thus, it could be expected that known downstream targets of MEF2C and RUNX2 transcription factors, like *ALPL* and *IBSP* genes, might also be affected, although they are not direct targets of miR-218. Indeed, in SaOS-2 cells, the gain of miR-218 resulted in significant reduction of ALP activity (Fig. 4a). Moreover, *IBSP* expression significantly dropped as well (Fig. 4b). *IBSP* is the major structural protein of the bone matrix, and its elevated expression is also associated with hypertrophy. In human cells, it has been shown to be regulated along with MEF2C, and not affected by RUNX2 [20].Therefore, miR-218 might regulate a whole cascade of hypertrophy-related factors during MSC chondrogenesis. To address this important question, we next assessed whether the effects caused by miR-218 in the osteosarcoma cell line model (SaOS-2) could be translated into human MSC during a course of chondrogenic differentiation.

### MiR-218 targets hypertrophic markers in MSC

**Fig. 5:**
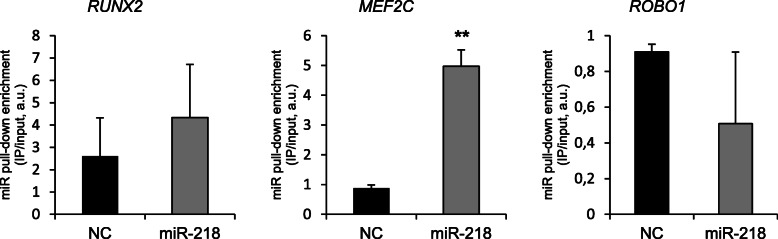
miR-218 targets hypertrophy-related genes in MSC. The miR pulldown assay was done in MSC (*N* = 2 donors). After cell lysis, biotinylated miR-218 mimic (gray) or a control non-targeting miR mimic (NC, black) was incubated with cytosolic fractions of the MSC cell extract, then immobilized and purified on magnetic streptavidin beads. After elution and total RNA extraction, abundancies of pulled-down mRNAs for indicated genes were measured using qRT-PCR and normalized to a housekeeping gene (*HPRT*). Mean values ± SD are shown; **p ≤ 0.01, two-tailed *t* test

To address the functional role of miR-218 in human MSC, we conducted a miR pulldown assay for a direct detection of miR-218 mRNA targets (Fig. 5). For this, miR-218 mimic labeled with biotin was incubated with the cytosolic fraction of a cellular extract of expanded human MSC, and using magnetic streptavidin beads, immobilized mRNA targets were isolated, purified, and analyzed. For the tested targets of miR-218: *MEF2C* and *RUNX2*, we detected high levels of enrichment in comparison to a non-targeting NC control miR mimic. *COL10A1* remained untested since it is not expressed at day 0 of chondrogenesis in MSC. Interestingly, although *ROBO1* (Roundabout 1) was earlier reported as a miR-218 target in rheumatoid arthritis (RA)-fibroblast-like synovial cells (FLS) [43], in human MSC, no interaction between miR-218 and *ROBO1* was detected (Fig. 5). Similarly, there was no enrichment for *HPGD* that earlier was reported to be a direct target of miR-218 in rabbit synovium-derived MSC [30] (data not shown). Collectively, these data emphasize that specific mRNA targets of miR-218 might also depend on a particular cellular context or a selected species.

### Effect of miR-218 on chondral versus endochondral differentiation of MSC

**Fig. 6:**
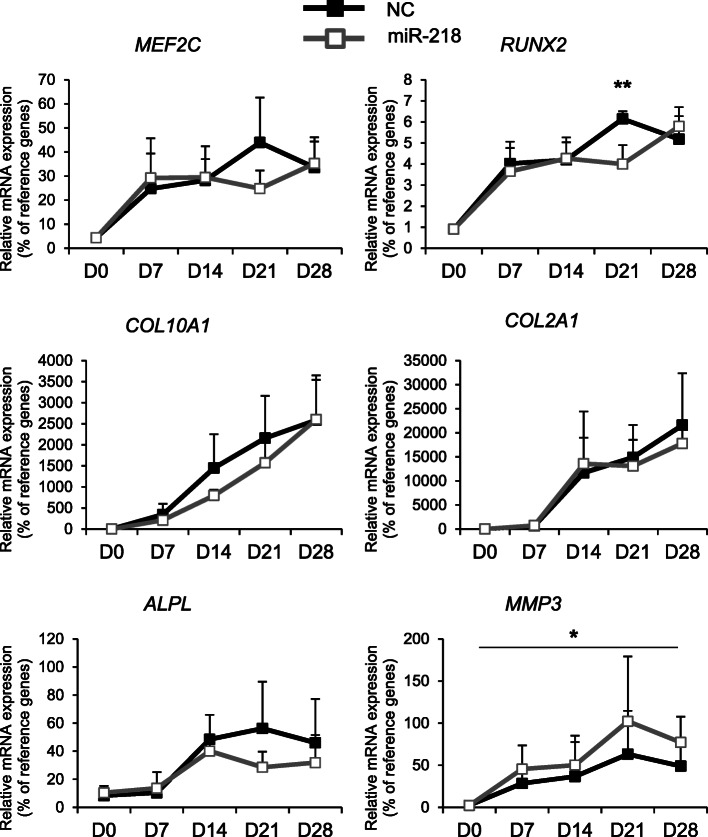
miR-218 reduces expression of hypertrophy-related markers during chondrogenesis of MSC. MSC (*N* = 4–5 donors) were transfected with miR-218 (gray), or with a control non-targeting miR (NC, black) mimic at day 0 and subjected to chondrogenic differentiation for 28 days; mRNA expression for indicated genes was monitored at indicated time points by qRT-PCR. Levels of mRNA expression were normalized to a geomean of HPRT, *CPSF6*, and *RPL13*; mean values ± SD are shown; *p ≤ 0.05, **p ≤ 0.01; NC vs miR281; two-tailed *t* test

To investigate whether miR-218 affects hypertrophy during MSC chondrogenesis, we transfected the cells with miR-218 mimic and followed-up the expression levels of hypertrophic markers during 28 days of a chondrogenic differentiation time course (Fig. 6 and Fig. S2). First, we confirmed that significantly elevated levels of miR-218 mimic persist over the 28 days’ time course of chondrogenic differentiation (Fig. S2). This also caused significant reduction of *RUNX2* expression by day 21 in MSC transfected with miR-218 in comparison to NC mimic control (Fig. 6). We also found that expression levels of *MEF2C*, *COL10A1*, and *ALPL* were decreased by the trend by day 21, with no apparent changes in *COL2A1* gene expression. Remarkably, these effects were accompanied by significant elevation of *MMP3* expression, indicating that although miR-218 caused reduction of several hypertrophic markers, a feedback loop might have been activated leading to induction of metalloproteinase *MMP3* expression (Fig. 6).

Having observed some inhibitory effects on hypertrophy by gain of miR-218, we were also interested to follow whether progression of chondrogenesis changed. Safranin O staining revealed only a slight trend for less proteoglycan deposition by MSC-derived chondrocytes at day 28 post-transfection with miR-218 in comparison to the non-targeting (NC) mimic transfection control (Fig. S3a). No apparent changes in GAG/DNA content were observed in both groups, with only a slight trend for reduced glycosaminoglycan deposition in case of MSC transfected with miR-218 (Fig. S3b).

Collectively, these data suggest that, although miR-218 had the ability to target the tested hypertrophic markers, it did not result either in significant reduction of the hypertrophic phenotype, or in an additional boost of chondrogenesis. Thus, no positive effect on a transition from endochondral towards chondral outcome during MSC chondrogenesis could be achieved when only this single miR was manipulated.

**Fig. 7:**
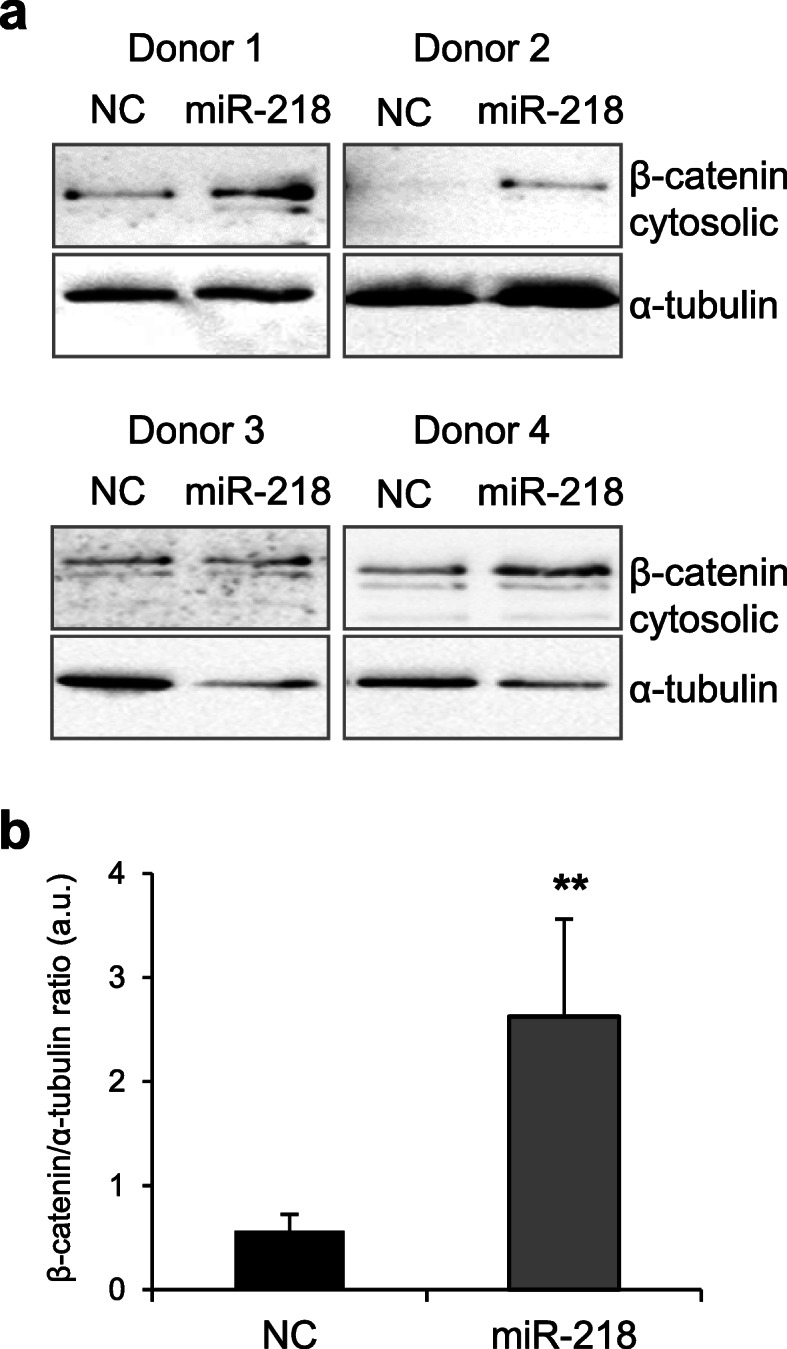
Gain of miR-218 at the start of chondrogenic differentiation leads to increased accumulation of cytosolic β-catenin. MSC (*N* = 4 donors) were transfected with miR-218 (gray), or with a control non-targeting miR (NC, black) mimic at day 0, subjected to chondrogenic differentiation for 28 days, and β-catenin accumulation (**a**) was assessed by Western blot analysis at day 28. **b** Semi-quantitative analysis of WB images; α-tubulin was used as a loading control; mean values ± SD are shown; *N* = 4; **p ≤ 0.01, two-tailed *t* test

Since it has been established before that regulation of both MEF2C [20] and RUNX2 [44] is WNT/β-catenin signaling-dependent, we were interested to find out whether ectopic overexpression of miR-218 might also cause alterations to WNT signaling. Indeed, we found that gain of miR-218 resulted in accumulation of active cytosolic β-catenin by day 28 of MSC chondrogenesis (Fig. 7), thus indicating that miR-218 might also target some of the WNT signaling inhibitors. According to an earlier report, miR-218 could activate canonical WNT signaling in mouse cells due to targeting *DKK2* and *SFRP2* [45]. Both DKK2 and SFRP2 are modulators of WNT signaling and could function as agonists or antagonists, depending on the cellular context and the presence of their co-factors (e.g., Kremen 2, in case of DKK2) [46, 47]. It is known that WNT/β-catenin signaling largely contributes to MSC hypertrophy [11]. Therefore, miR-218 may not only inhibit hypertrophic markers but also induce a feedback loop that restores hypertrophy due to concomitant activation of WNT/β-catenin signaling.

### Expression of miR-218 in cartilage of OA patients

**Fig. 8:**
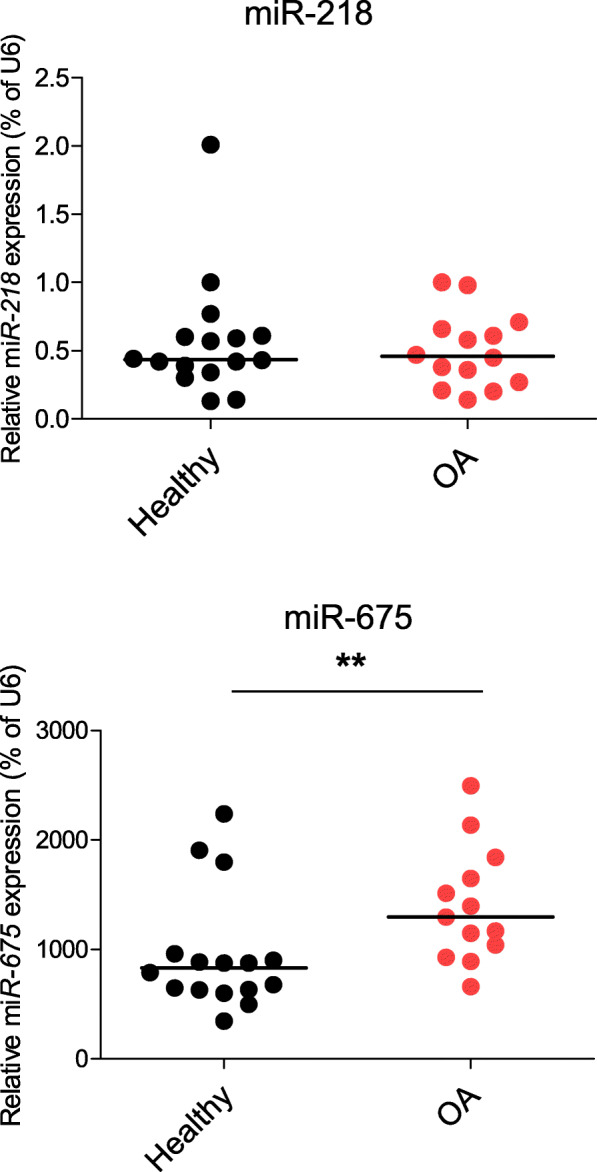
There are no differences in miR-218 expression levels between healthy and OA cartilage. Levels of expression for indicated miRs were measured in cartilage tissue isolated from 16 healthy and 13 osteoarthritis (OA) patients by qRT-PCR and normalized to U6. Horizontal lines are set at median values, **p ≤ 0.01 (Mann-Whitney *U*)

Finally, as hypertrophic degeneration of chondrocytes is one of the major characteristics of OA development, we hypothesized that miR-218 might be differentially expressed between healthy and pathological phenotypes of cartilage tissue. We assessed expression levels of miR-218 in healthy donors and OA patients (Fig. 8). It is important to mention that all the cartilage samples were additionally stratified by levels of miR-675 expression, a reliable marker of OA progression in our hands [37, 48]. Indeed, healthy and OA patient cohorts were well-segregated due to significantly different levels of miR-675 expression. However, no significant differences between chondrocytes from healthy donors and OA patients were observed for miR-218 expression. Thus, in our tested cohorts, we could not confirm a conclusion made by a previous report claiming that miR-218 is a prognostic marker and a therapeutic target for OA treatment [49].

**Fig. 9:**
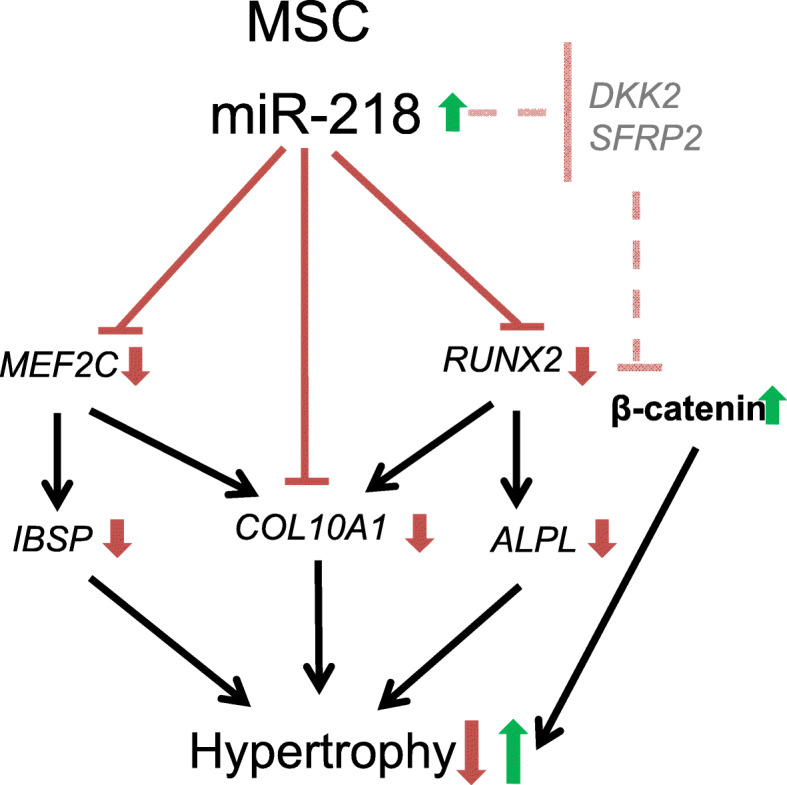
A scheme depicting a putative mechanism for the anti-hypertrophic effects caused by miR-218 in MSC and activation of a feedback loop due to concomitant effects on inhibitors of WNT/β-catenin signaling. MiR-218 targets hypertrophy-related mRNAs: *MEF2C* and its downstream targets, *COL10A1* and *IBSP*, leading to attenuation of the hypertrophic phenotype. Among the other targets of miR-218, there is *RUNX2*, a transcription factor regulating expression of *COL10A1* and *ALPL*. However, accumulation of β-catenin due to concomitant targeting of WNT signaling inhibitors activates a feedback loop that counteracts the anti-hypertrophic alterations. Arrows indicate directions of expression regulation: red—downregulation, green—upregulation

Altogether, our data suggest that anti-hypertrophic miR-218 affects a number of important genes within a hypertrophic differentiation cascade (Fig. 9). While miR-218 targeted hypertrophy-related mRNAs, such as *MEF2C* and its downstream targets *COL10A1* and *IBSP*, as well as *RUNX2*, a transcription factor regulating the expression of *COL10A1* and *ALPL*, it also resulted in accumulation of β-catenin. WNT/β-catenin signaling was induced, probably, due to a concomitant targeting of WNT signaling inhibitors, such as *SFRP2* and *DKK2*, which in turn led to the activation of a feedback loop counteracting the anti-hypertrophic effects of miR-218. Therefore, one cannot exclude that other mRNA targets regulated by miR-218 might influence the final outcome of chondral versus endochondral progression, depending on a particular cellular context.

## Discussion

The cartilage tissue is characterized by a low cellular density and vascular components, and it is mostly comprised by proteoglycans and extracellular collagens. It has a very limited capacity to heal in case of injury or degeneration processes due to osteoarthritis. Therefore, therapeutic approaches with application of alternative cells sources, such as MSC that are capable of chondrogenic differentiation, are at a focus of orthopedic research. However, a long-standing problem with application of MSC for cartilage regeneration is their tendency to undergo hypertrophic degeneration that could lead to endochondral bone induction after transplantation. The most characteristic feature of hypertrophic chondrocytes is expression of type X collagen and ALP. These markers are absent in articular chondrocytes that retain the stable phenotype. This implies that the hypertrophic pathways promoted in MSC during chondrogenesis are completely blocked in AC due to some yet to be identified molecular mechanisms.

There is growing evidence that microRNAs are involved in chondrogenic differentiation of MSC. However, there are only a limited number of studies on a regulatory role of miRs in hypertrophy. To address a question about miRs’ role in driving hypertrophy, we conducted a comparative analysis of expression profiles for 1349 miRs between hypertrophic and articular chondrocytes. Our focus was on finding characteristic signature-miRs that would define these two different chondrocyte phenotypes. Among the top 15 differentially expressed miRs, we selected miR-218 that resembled a suitable candidate, as among its in silico predicted targets, there were well-characterized hypertrophy markers, such as *MEF2C*, *RUNX2*, and *COL10A1*.

Mir-218-5p is a microRNA found to be expressed in 20 vertebrate species (http://www.mirbase.org/). It has been implicated in tumorigenesis and cancer progression of different tumor entities (reviewed in [50]), as well as its role in the motor neuron development has recently been discovered [51, 52]. However, regarding the role of miR-218 in chondrogenesis, the published literature is very limited and so far presents somewhat contradicting claims. Namely, there is a single study conducted with application of rabbit synovium-derived MSC that concluded the pro-chondrogenic role of miR-218 in early chondrogenesis due to targeting *HPGD*, although a link between *HPGD* alterations and effects on chondrogenesis has not been established [30]. On the other hand, another study implied that elevated expression of miR-218 positively correlates with OA progression and cartilage tissue loss in patients due to targeting *PIK3C2A* of Akt pathway, thus contradicting a conclusion of the study mentioned above about pro-chondrogenic effects of miR-218 [49]. To summarize, no studies on the role of miR-218 in human MSC that would question whether there is a direct link between miR-218 and chondrocyte hypertrophy have been conducted to date.

It has been already found that miR-218 could target *Runx2* in mouse ACDT5 cell line, although this effect was found to be cell-type dependent [28]. Nevertheless, we used this finding as a positive example for evaluation of other putative targets of miR-218, *MEF2C* and *COL10A1*, and confirmed that all three genes were directly affected by an ectopic miR-218 mimic, as the corresponding reporters’ activity, gene expression, and protein accumulation were reduced. Since miR-218 was found to regulate not only one target related to hypertrophy but a number of genes of the hypertrophic cascade, including those that were not predicted to directly interact with miR-218, such as *IBSP* and *ALPL*, we concluded an anti-hypertrophic role of miR-218.

An important question, however, remained whether miR-218 could not only prevent hypertrophy but also promote chondral differentiation of human MSC as one might expect when the expression of anti-chondrogenic *RUNX2* [20] is reduced. Although we were able to confirm targeting of hypertrophic markers by miR-218 in human MSC, we did not achieve any significant anti-hypertrophic effects, as well as a hypothesis about its pro-chondrogenic role had to be rejected, as additional gain of miR-218 at start of differentiation failed to boost chondrogenic markers further. These results contradict the previous report about the ability of miR-218 to promote early chondrogenesis of rabbit MSC from synovial fluid [30]. However, we also argue against a suggested pro-osteogenic role of miR-218 due to targeting of *ROBO1* reported for rheumatoid arthritis FLS [43]. Indeed, we did not detect interaction between *ROBO*1 and the miR-218 bait in MSC, probably, due to different representation of multiple putative mRNA targets present at a given cell type. It also implies that the same miR might play different regulatory functions depending on a particular cellular context. We also found that, although miR-218 reduced hypertrophic mRNAs by trend, it may also target genes that in turn induce pro-hypertrophic pathways, like WNT/β-catenin signaling, thus generating a feedback loop that prevents a shift towards a stable chondrogenic phenotype. Therefore, to achieve a full inhibition of the endochondral pathway and successful outcome of chondral development of human MSC, not one, but a whole class of anti-hypertrophic miRs, including miR-218, needs to be taken into consideration for tissue engineering purposes. Finding these additional factors that are able to control chondrogenic differentiation and prevent hypertrophy would be of interest in future investigations.

Next, we also speculated that miR-218 might serve as a marker to distinguish between the healthy and pathological phenotype of chondrocytes in OA, as the upregulation of hypertrophic markers in chondrocytes is one of the main characteristics of OA cartilage [37, 53]. In contrary to a recent report suggesting that an upregulation of miR-218 is a characteristic of cartilage destruction in OA patients [49], no substantial differences between healthy and OA cartilages were found for miR-218 within our experimental cohorts. Importantly, for stratification of cartilage samples, we additionally applied expression of miR-675 a decisive factor to verify segregation of healthy and OA cohorts, as miR-675 has been reported to be a reliable marker for OA progression [37, 48]. Nevertheless, even without this stratification, in our larger samples sets, no differential expression between healthy donors and OA patients cohort was found. This highlights that a conclusion about miR-218 as a prognostic marker and a therapeutic target for OA treatment has to be taken with precaution.

## Conclusions

In conclusion, our study suggests that miR-218 contributes to a balance between endochondral versus chondral differentiation in human MSC as it targets a number of important genes regulating the hypertrophic pathway. While miR-218 suppresses *MEF2C*, *RUNX2*, and *COL10A1* that define the terminal differentiation of chondrocytes, it may also reduce other genes inhibiting the pro-hypertrophic WNT/β-catenin pathway, thus activating a feedback loop. Therefore, with a single miR, one cannot achieve a challenging goal of a phenotype switch from endochondral to chondral, and a whole class of hypertrophy-regulating miRs have to be taken into consideration. These findings give new insights into mechanisms of hypertrophic differentiation and chondrogenesis in human MSC and offer a perspective for further investigations into understanding mechanisms regulating cartilage disorders.

## Supplementary Information

**Additional file 1: Supplementary Table S1.** List of qRT-PCR primers used in this study. **Supplementary Table S2.** List of cloning primers used in this study. **Fig. S1.** miR-218 transfection efficiency in SaOS-2 cells. SaOS-2 cells were transiently transfected with miR-218 (grey), or with non-targeting control (NC, black) mimic, and transfection efficiency was controlled by qRT-PCR. Mean values ± SD are shown, *n* = 2; ***p ≤ 0.001, two-tailed t-test. **Fig. S2.** miR-218 transfection efficiency in human MSC. MSC cells (*N* = 3 donors) were transiently transfected with miR-218 (grey), or with non-targeting control (NC, black) mimic before cells were subjected to chondrogenic differentiation for 28 days, and transfection efficiency was controlled by qRT-PCR at indicated time points. Mean values ± SD are shown; *p ≤ 0.05, NC vs miR-218; expression kinetic curves were compared using two-tailed paired t-test. **Fig. S3.** Gain of function of miR-218 at the start of chondrogenic differentiation does not additionally boost MSC chondrogenesis. MSC (*N* = 4 - 6) were transfected with miR-218 (grey), or with a control non-targeting miR (NC, black) mimics at day 0, and subjected to chondrogenic differentiation for 28 days. a, Safranin O staining of 3D pellet cultures at day 28 chondrogenesis. Scale bar, 500 μm. b, Total GAG and DNA content of 3D pellet cultures was assessed by DMMB and PicoGreen assays at day 28 of chondrogenesis; Mean values ± SD are shown; *N* = 6 donors.

## Data Availability

The datasets generated during and/or analyzed during the current study are available from the corresponding author on reasonable request.

## Abbreviations

AC: Articular chondrocytes
ATCC: American Type Culture Collection
ALP: Alkaline phosphatase
BMP: Bone morphogenetic protein
DAVID: The Database for Annotation, Visualization and Integrated Discovery
DSC: Dental stem cells
DMMB: Dimethyl-methylene blue
EDTA: Ethylenediaminetetraacetic acid
FCS: Fetal calf serum
FGF: Fibroblast growth factor
GO: Gene ontology
HBV: Hepatitis B virus
HCV: Hepatitis C virus
HIV: Human immunodeficiency virus
IGF: Insulin-like growth factor
IHH: Indian hedgehog
MAD: Median of absolute deviation
MSC: Mesenchymal stromal cells
miR: MicroRNA
OA: Osteoarthritis
qRT-PCR: Quantitative reverse-transcriptase polymerase chain reaction
PBS: Phosphate-buffered saline
PTHrP: Parathyroid hormone-related protein
mirSVR: microRNA score of vector regression
TGFβ: Transforming growth factor beta
3’UTR: 3′-Untranslated region
WB: Western blotting
