# Supplementary material for: MiR-218 affects hypertrophic differentiation of human mesenchymal stromal cells during chondrogenesis via targeting RUNX2, MEF2C, and COL10A1
Source: Stem Cell Res Ther. 2020 Dec 10;11:532. doi: 10.1186/s13287-020-02026-6 (PMC7727242; doi:10.1186/s13287-020-02026-6)
Supplement: Supplementary file 1 — Additional file 1: Supplementary Table S1. List of qRT-PCR primers used in this study. Supplementary Table S2. List of cloning primers used in this study. Fig. S1. miR-218 transfection efficiency in SaOS-2 cells. SaOS-2 cells were transiently transfected with miR-218 (grey), or with non-targeting control (NC, black) mimic, and transfection efficiency was controlled by qRT-PCR. Mean values ± SD are shown, n = 2; ***p ≤ 0.001, two-tailed t-test. Fig. S2. miR-218 transfection efficiency in human MSC. MSC cells (N = 3 donors) were transiently transfected with miR-218 (grey), or with non-targeting control (NC, black) mimic before cells were subjected to chondrogenic differentiation for 28 days, and transfection efficiency was controlled by qRT-PCR at indicated time points. Mean values ± SD are shown; *p ≤ 0.05, NC vs miR-218; expression kinetic curves were compared using two-tailed paired t-test. Fig. S3. Gain of function of miR-218 at the start of chondrogenic differentiation does not additionally boost MSC chondrogenesis. MSC (N = 4 - 6) were transfected with miR-218 (grey), or with a control non-targeting miR (NC, black) mimics at day 0, and subjected to chondrogenic differentiation for 28 days. a, Safranin O staining of 3D pellet cultures at day 28 chondrogenesis. Scale bar, 500 μm. b, Total GAG and DNA content of 3D pellet cultures was assessed by DMMB and PicoGreen assays at day 28 of chondrogenesis; Mean values ± SD are shown; N = 6 donors. [file 13287_2020_2026_MOESM1_ESM.pdf]

Electronic supplementary material for:

MiR-218 affects hypertrophic differentiation of human mesenchymal stromal cells during chondrogenesis *via* targeting *RUNX2, MEF2C,* and *COL10A1*

Svitlana Melnik, Jessica Gabler, Simon Dreher, Nicole Hecht, Nina Hofmann, Tobias Großner, Wiltrud Richter

To whom correspondence should be addressed:

E-mail: wiltrud.richter@med.uni-heidelberg.de

This pdf file includes:

Supplementary Tables S1 – S2
Supplementary Fig. S1 – S3

Supplementary Table S1 List of qRT-PCR primers used in this study

Gene Name	Sequence
ACTB	CTCTTCCAGCCTTCCTTCCT<br>CGATCCACACGGAGTACTTG
ALPL	CACCAACGTGGCTAAGAATG<br>ATCTCCAGCCTGGTCTCCTC
AXIN2	TAGCTGAGGTGTCGAAGCC<br>CCTTCAGCATCCTCCGGTAT
COL10A1	TTGCTCTCCTCTTACTGCTAT<br>TTACGCTGAACGATACCAAA
COL2A1	TGGCCTGAGACAGCATGAC<br>AGTGTTGGGAGCCAGATTGT
HPRT	AAGGGTGTTTATTCCTCATGGA<br>CCTCCCATCTCCTTCATCAC
CPSF6	AAGATTGCCTTCATGGAATTGAG<br>TCGTGATCTACTATGGTCCCTCTCT
MEF2C	GTATGGCAATCCCCGAAACT<br>ATCGTATTCTTGCTGCCTGG
RUNX2	ACTCTACCACCCCGCTGTC<br>CAGAGGTGGCAGTGTCATCA
RLP13	CATTTCTGGCAATTTCTACAG<br>CAGGCAACGCATGAGGAAT
ROBO1	AGTGACGCTGGCAAATATGT<br>CTGCCAAGTTACTGGGTCTC

Supplementary Table S2 List of cloning primers used in this study

Gene Name	Sequence
MEF2C	AAACTAGTCCCATCCTTACAACCCATCCT<br>CGCAAGCTTTGTTACTCTAGGACGGGTTATGT
COL10A1	AAACTAGTGCTGAGCCTTGTGCCTCCCATTCAAGT<br>GCGCGCAAGCTTAGCCTTGAAAGAATGGTTGAGA

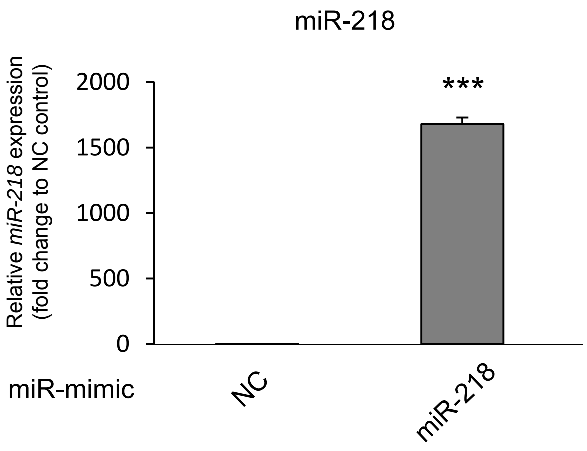

Fig. S1 miR-218 transfection efficiency in SaOS-2 cells. SaOS-2 cells were transiently transfected with miR-218 (grey), or with non-targeting control (NC, black) mimic, and transfection efficiency was controlled by qRT-PCR. Mean values ± SD are shown, n = 2; ***$p \leq 0.001$, two-tailed *t*-test.

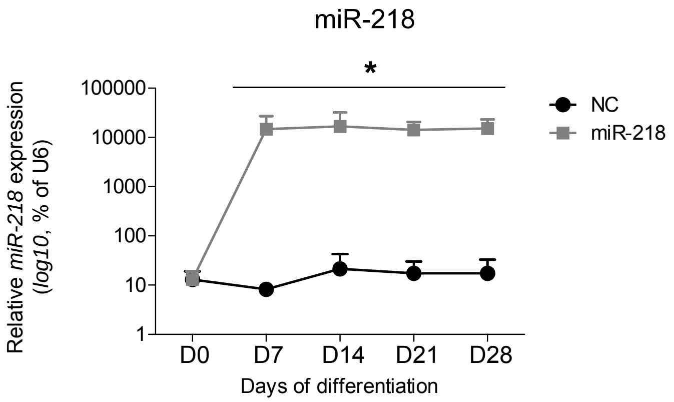

Fig. S2 miR-218 transfection efficiency in human MSC. MSC cells (N = 3 donors) were transiently transfected with miR-218 (grey), or with non-targeting control (NC, black) mimic before cells were subjected to chondrogenic differentiation for 28 days, and transfection efficiency was controlled by qRT-PCR at indicated time points. Mean values ± SD are shown; *$p \leq 0.05$, NC *vs* miR-218; expression kinetic curves were compared using two-tailed paired *t*-test.

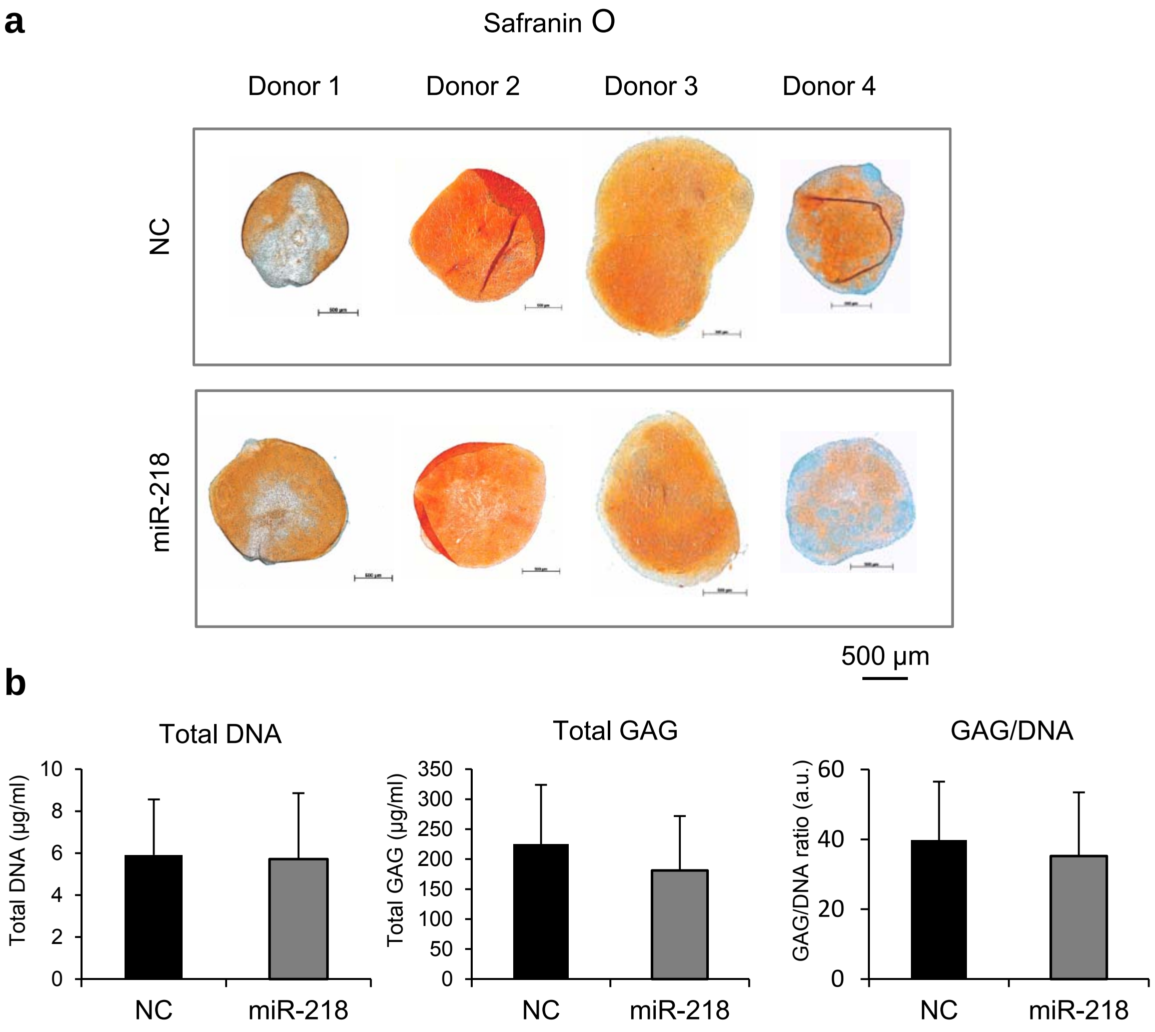

Fig. S3 Gain of function of miR-218 at the start of chondrogenic differentiation does not additionally boost MSC chondrogenesis. MSC (N = 4 - 6) were transfected with miR-218 (grey), or with a control non-targeting miR (NC, black) mimics at day 0, and subjected to chondrogenic differentiation for 28 days. **a,** Safranin O staining of 3D pellet cultures at day 28 chondrogenesis. Scale bar, 500 µm. **b,** Total GAG and DNA content of 3D pellet cultures was assessed by DMMB and PicoGreen assays at day 28 of chondrogenesis; Mean values ± SD are shown; N = 6 donors.
